# Beyond Painful Bones and Renal Stones: Acute Crystal Arthropathy of Dual Aetiology May Be an Unforeseen Complication Following Parathyroid Surgery

**DOI:** 10.7759/cureus.107277

**Published:** 2026-04-18

**Authors:** Amy Sara Varghese, Rebecca John, Thomas Paul, Paul M Jacob, Elanthenral Sigamani, Kripa Cherian

**Affiliations:** 1 Endocrinology, Diabetes and Metabolism, Christian Medical College, Vellore, Vellore, IND; 2 Endocrine Surgery, Christian Medical College, Vellore, Vellore, IND; 3 Pathology, Christian Medical College, Vellore, Vellore, IND

**Keywords:** acute crystal arthropathy, calcium pyrophosphate dihydrate disease, gout, gout crystals, gout flare, primary hyperparathyroidism, pseudogout

## Abstract

Primary hyperparathyroidism (PHPT), one of the most common endocrine conditions, has myriad manifestations, including nephrolithiasis, osteoporosis, neuropsychiatric complications and fatigue. The treatment of choice is focused parathyroidectomy when the lesion is localised. Common complications following parathyroidectomy include hypoparathyroidism and hungry bone syndrome. Acute crystal arthropathy due to gout and pseudogout following parathyroid surgery is scarcely reported. We report the case of a 47-year-old man who developed painful swelling of bilateral ankle joints following parathyroidectomy for PHPT. Synovial fluid aspirate from the ankle joint showed positively birefringent rhomboid-shaped calcium pyrophosphate dihydrate (CPPD) crystal and needle-shaped crystal of mono-sodium urate on polarised light microscopy. He was initiated on analgesics and glucocorticoids and had symptomatic improvement. The development of acute painful swelling of the joints following parathyroidectomy should alert the clinician to the possibility of gout and pseudogout; the timely recognition and treatment will aid in quick recovery.

## Introduction

Primary hyperparathyroidism (PHPT) is among the most common endocrine diseases encountered. The classical presentation of primary hyperparathyroidism may be described by Albright’s pentad of “painful bones, renal stones, abdominal groans, psychic moans and fatigue overtones” [1,2]. However, beyond the pentad, PHPT may have diverse presentations and complications. PHPT may be associated with hypertension, a heightened cardiovascular risk, and, in some cases, hyperuricemia, which can lead to gout [3]. Common complications following successful parathyroid surgery include hypoparathyroidism, characterised by low serum calcium, elevated serum phosphate and low parathyroid hormone (PTH) levels, and in select circumstances, a hungry bone syndrome characterised by severe hypocalcaemia and hypophosphataemia. Acute crystal arthropathy refers to an acute inflammatory arthritis caused by the deposition of crystals within a joint, leading to pain and swelling of the affected joint. This could be a result of precipitation of monosodium urate crystals within the synovial space in the setting of hyperuricaemia (gout) or the accumulation of calcium pyrophosphate dihydrate (CPPD) crystals (pseudogout) [4]. Acute crystal arthropathy manifesting after parathyroid surgery, though reported in literature, is not commonly encountered in the clinical scenario. Here, we present the case of a 47-year-old man who presented with painful joint swelling following parathyroid surgery and was diagnosed with an acute crystal arthropathy secondary to both gout and pseudogout.

## Case presentation

**Figure 1:**
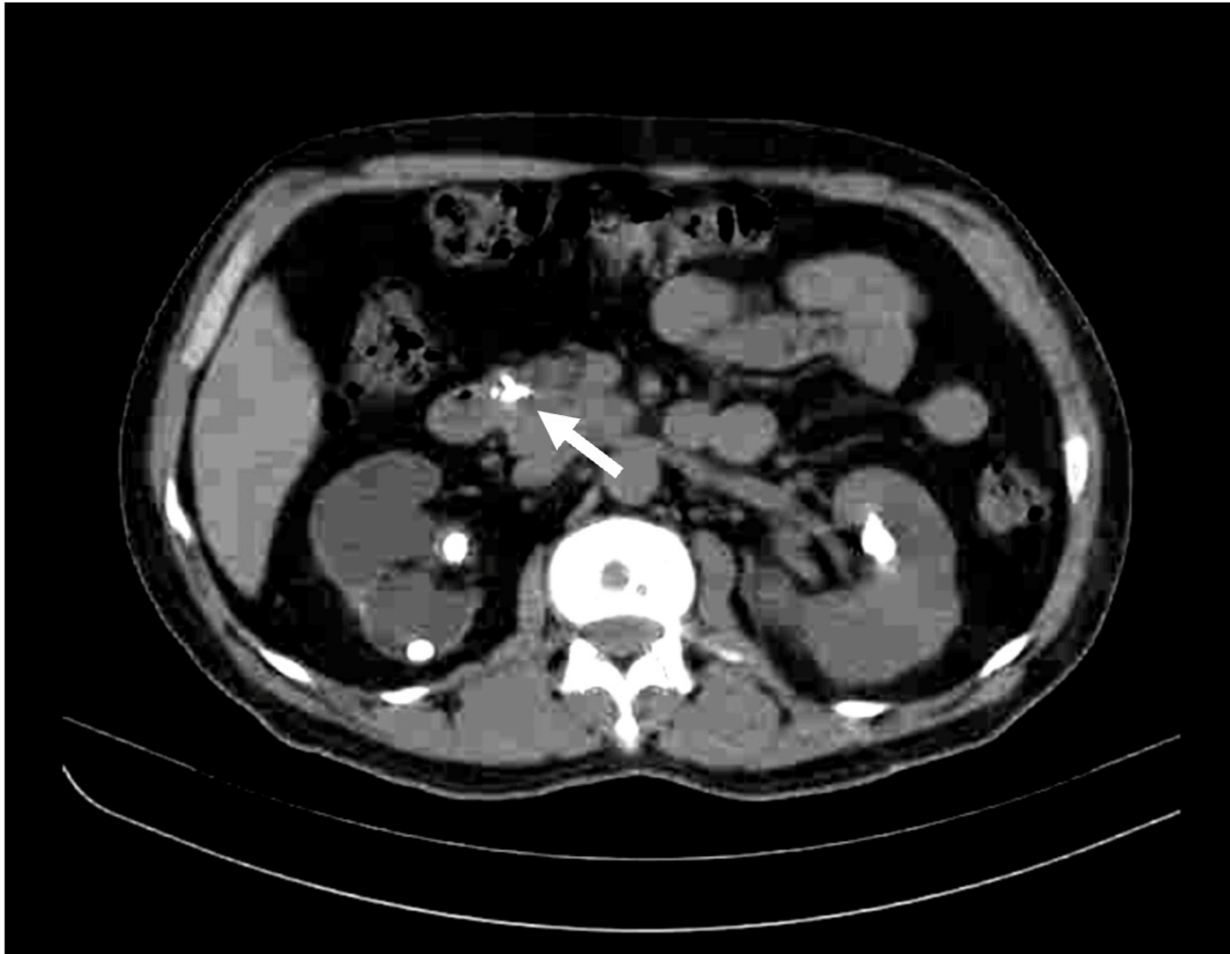
CT scan of the pancreas revealed chunky calcification (arrow) in the head of the pancreas. Calcification of the pancreas is an expected complication of long-standing hypercalcemia and may result in both exocrine and endocrine insufficiencies.

**Figure 2:**
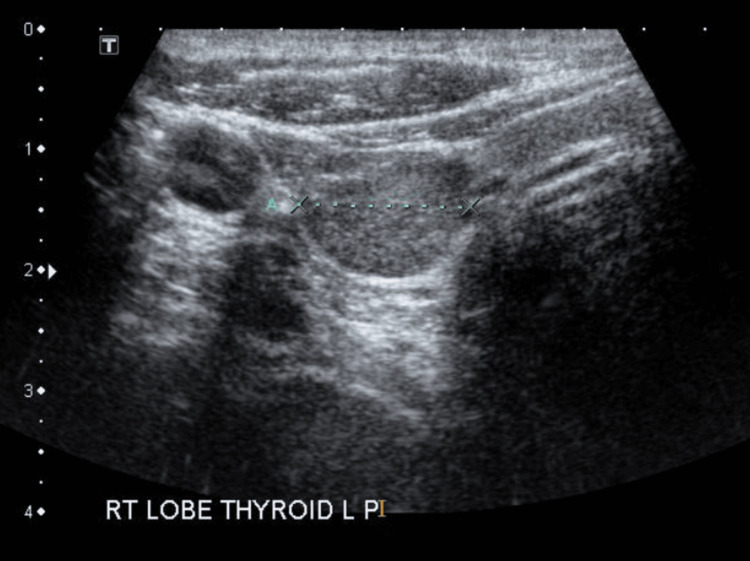
USG of the neck confirmed a right inferior parathyroid adenoma. Parathyroid adenomas on USG are seen as oval or bean-shaped, well-defined and hypoechoic masses located posterior to the thyroid gland. They are highly vascular on Doppler imaging.

**Figure 3:**
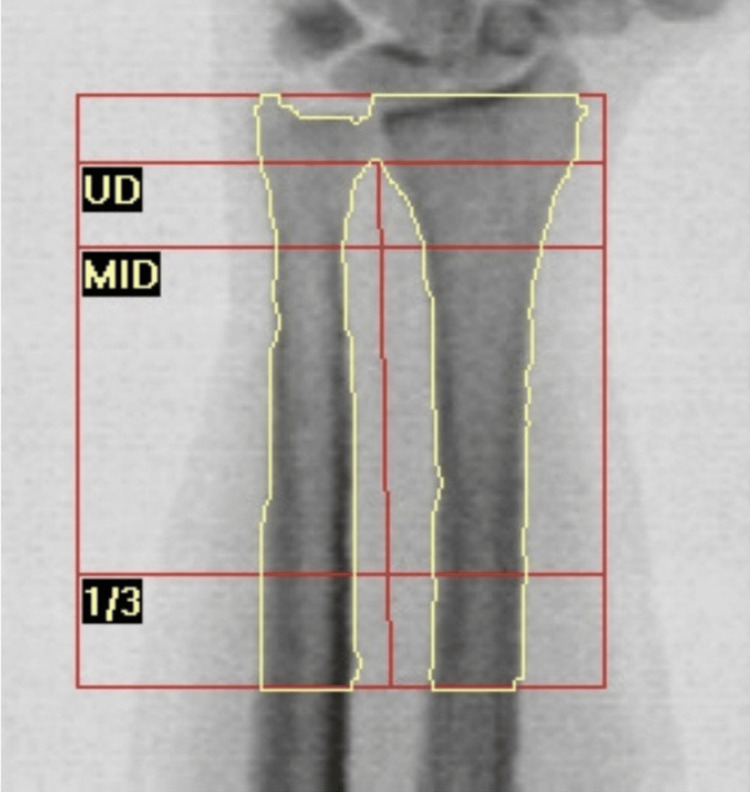
Dual-energy X-ray absorptiometry scan demonstrated severe osteoporosis at the distal forearm with a T-score of -3.5. Primary hyperparathyroidism characteristically shows predominant involvement of the distal forearm, owing to the high proportion of cortical bone at this site.

**Figure 4:**
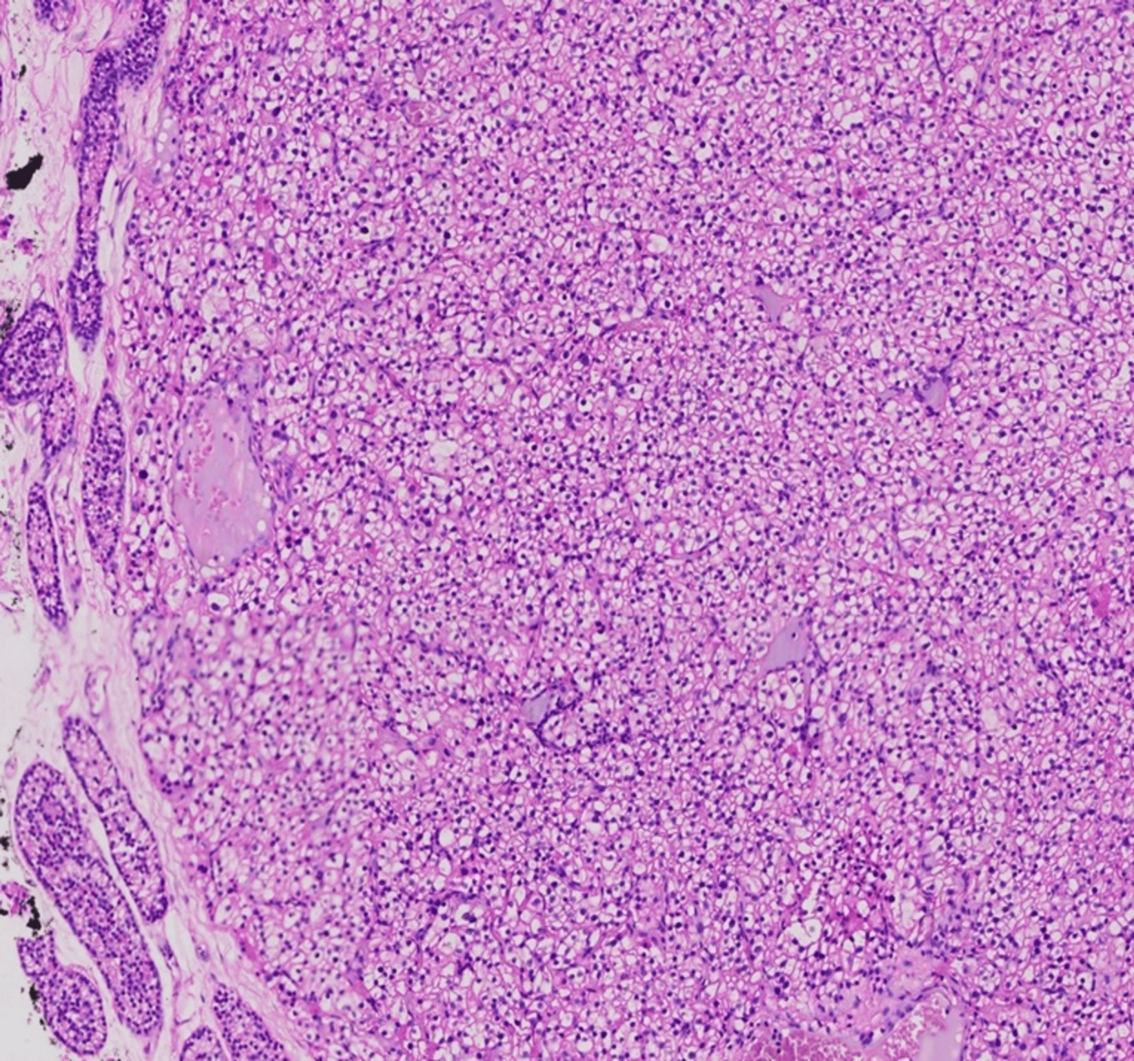
Histopathologic examination of the surgical specimen demonstrated a well-circumscribed tumour with compressed normal parathyroid tissue (H&E, 100×). The circumscribed parathyroid tumour is composed of closely arranged nests, trabeculae and sheets of polygonal cells with mild pleomorphic nuclei, granular chromatin, inconspicuous nucleoli and moderate amounts of pale eosinophilic cytoplasm. The tumour nests are separated by thin-walled vascular channels. Mitotic activity is inconspicuous. There is no evidence of capsular invasion, vascular invasion or nuclear atypia.

**Figure 5:**
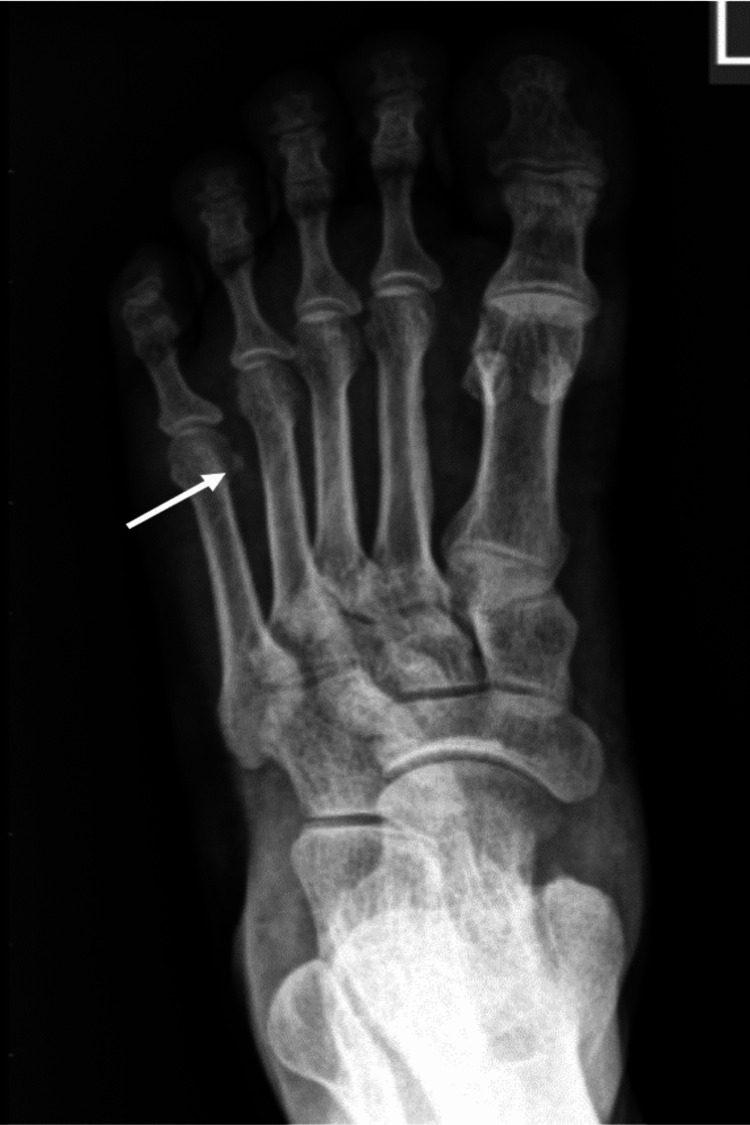
X-ray of the left foot showed chondrocalcinosis (arrow). Chondrocalcinosis appears on conventional radiograph as linear or punctate deposits (calcification) within the cartilage of the affected joint and is suggestive of pseudogout (calcium pyrophosphate deposition disease).

**Figure 6:**
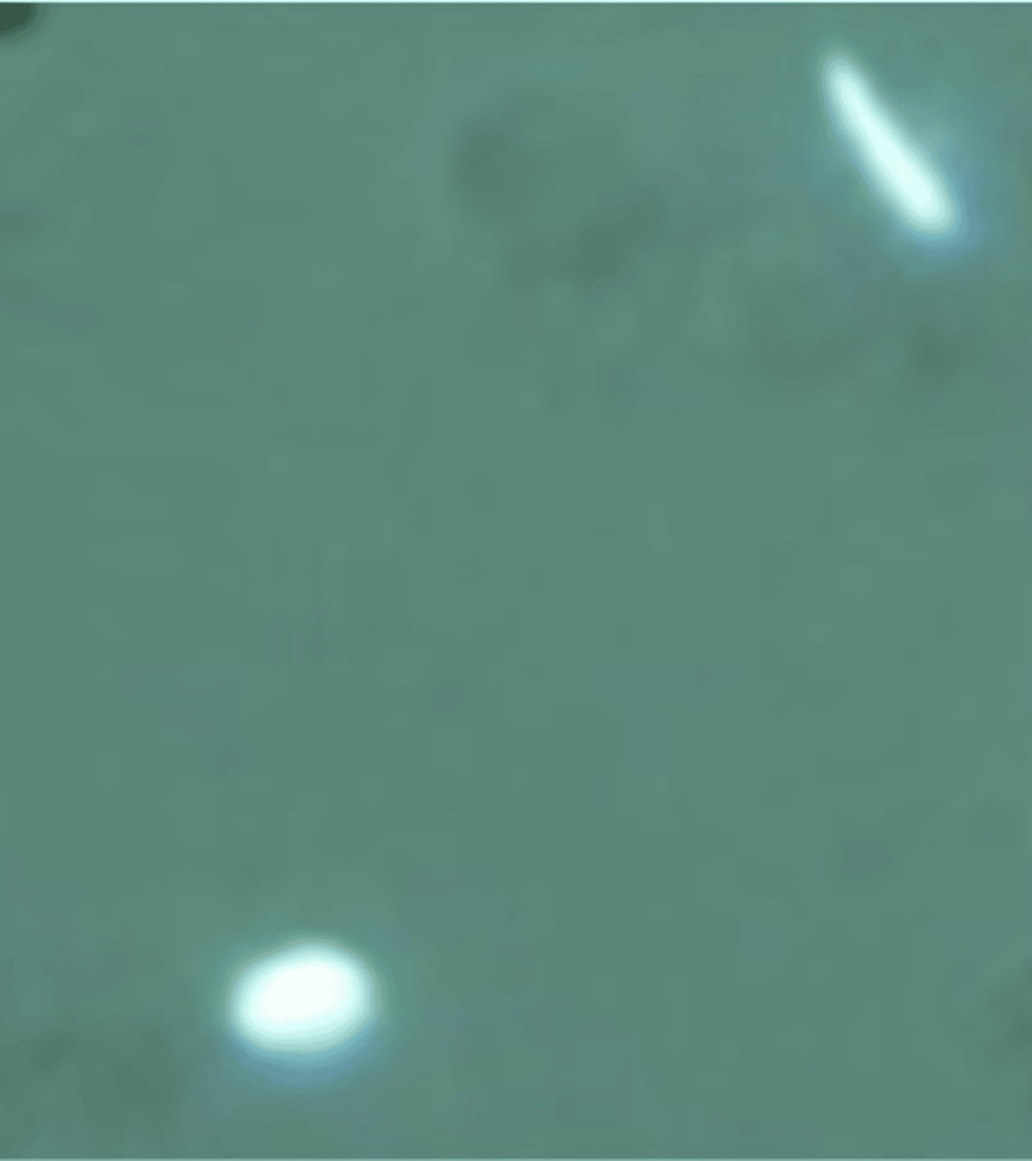
Synovial fluid aspirate showed the presence of rhomboid-shaped calcium pyrophosphate dihydrate (CPPD) crystal and needle-shaped crystal of sodium mono-urate sodium on microscopy. Uric acid crystals are typically negatively birefringent and appear yellow on polarised microscopy when parallel to the compensator axis. In comparison, CPPD crystals are positively birefringent and appear blue in colour when parallel to the compensator axis.

A 47-year-old man presented with complaints of polyuria for six months. He had a history of recurrent renal calculi with a history of passing stones in urine. There was no history of hypertension, diabetes mellitus or cardiovascular diseases. There was no similar history in the family. His systemic examination was unremarkable. Evaluation of these symptoms revealed a staghorn calculus at the right pelvi-ureteric junction with gross hydroureteronephrosis. Biochemical evaluation revealed an elevated albumin-adjusted calcium of 14 mg/dL with an unsuppressed parathormone (PTH), which was more than 25 times the upper limit of normal. Thus, he was diagnosed with a PTH-dependent hypercalcemia. He had normal serum phosphate; however, tubular maximum phosphate reabsorption per glomerular filtration rate (TmP/GFR) was 1.1, indicating renal phosphate wasting. Vitamin D insufficiency was noted, as well as an elevated serum alkaline phosphatase, which indicates active bone involvement and a high risk of postoperative hungry bone syndrome. His serum creatinine was elevated (1.8 mg/dL), and hyperuricemia (11.9 mg/dL) was noted preoperatively. The biochemical parameters have been depicted in Table 1. Calcific deposits were noted in the pancreas, although there was no evidence of exocrine or endocrine insufficiency (Figure 1). Calcific deposits are known to occur in prolonged hypercalcemia and can present with recurrent pancreatitis or sequelae of chronic pancreatitis. Localisation studies confirmed the presence of a right inferior parathyroid lesion that was concordant on parathyroid scintigraphy and sonogram of the neck (Figure 2). Bone mineral density assessment by dual-energy X-ray absorptiometry (DXA) revealed severe osteoporosis at the forearm, which is typical of hyperparathyroidism due to the predominance of cortical bone at the site (Figure 3). The DXA parameters are presented in Table 2. Hypercalcaemia was managed with hydration, and he was started on febuxostat 40 mg once daily for the hyperuricemia, and uric acid had normalised to 3 mg/dL at the time of surgery. He underwent focused right inferior parathyroidectomy. The biopsy of the specimen was reported as a parathyroid adenoma with no features of nuclear atypia, capsular or vascular invasion (Figure 4). Following surgery, he developed symptomatic hypocalcaemia with hungry bone syndrome, which was treated with calcium and calcitriol supplements. On the third postoperative day, he developed an acute onset of severe pain and swelling involving the bilateral ankle joint, which was gradually progressive and affected his mobility. There was no documented fever. Examination revealed swollen, tender, erythematous ankle joints bilaterally. Inflammatory markers were elevated, with erythrocyte sedimentation rate (ESR) being 99 mm/hour and CRP being 42.8 mg/dL (N: < 6 mg/dL), suggestive of acute inflammation. His repeat serum uric acid was 4.2 mg/dL. X-ray of the left foot showed chondrocalcinosis, a finding suggestive of calcifications within the cartilage of a joint and indicative of pseudogout (Figure 5). Ultrasound examination confirmed the presence of fluid in the tibio-talar joint with chondrocalcinosis. A synovial fluid aspirate showed the presence of rhomboid-shaped CPPD crystals and needle-shaped crystals of sodium mono-urate sodium on microscopic examination (Figure 6). Uric acid crystals are typically negatively birefringent and appear yellow on polarised microscopy when parallel to the compensator axis, while CPPD crystals are positively birefringent and appear blue in colour when parallel to the compensator axis. This confirmed the presence of both gout and CPPD disease contributing to his acute crystal arthritis. He was initiated on analgesics and tablet deflazacort 12 mg once a day, with significant improvement in his pain and swelling over the next 10 days. His steroids were gradually tapered over the next two months with continuation of febuxostat.

**Table 1:** Biochemical parameters of the patient. eGFR, estimated glomerular filtration rate; TmP/GFR, tubular maximum phosphate reabsorption per glomerular filtration rate

Variable	Value	Reference range
Serum calcium (mg/dL) (corrected for albumin)	14.6 (4.0)	8.3-10.4
Serum phosphate (mg/dL)	3.3	2.5-4.6
Serum parathyroid hormone (pg/mL)	>1900	8-74
Serum creatinine (mg/dL) (eGFR, mL/min/1.73 m²)	1.81 (44.48)	0.5-1.4
Serum 25(OH) vitamin D (ng/mL)	26.18	>30
Serum alkaline phosphatase (U/L)	715	40-125
Urine calcium excretion (mg/kg/24 hours)	4.6	<4
TmP/GFR	1.1	>2.5
Serum uric acid (mg/dL)	11.9	4-7

**Table 2:** Bone mineral density parameters of the subject.

Site	Bone mineral density (g/cm²)	T-score	Z-score
Lumbar spine	0.962	-1.3	-1.0
Neck of femur	0.658	-2.0	-1.3
Distal radius	0.607	-3.8	-3.5

## Discussion

Primary hyperparathyroidism may present with varied musculoskeletal complications, which include pseudogout, vertebral fracture, myopathy, and cord compression [5]. Pseudogout, a rare sequelae described following parathyroidectomy, is an acute episode of calcium pyrophosphate deposit-induced synovitis resulting from disturbances in calcium-phosphate metabolism [6]. It is often associated with hungry bone syndrome, a well-known postoperative phenomenon of rapid, profound and prolonged hypocalcaemia associated with hypophosphatemia and hypomagnesaemia. The likelihood of hungry bone syndrome is increased among those with preoperative severe bone disease, like that seen in our patient with PTH around 25 times the upper limit of normal and elevated ALP. It most frequently manifests within 24-48 hours post-parathyroidectomy. The most frequently affected joints include the knees, followed by shoulders, wrists and ankles [7]. In our patient, bilateral ankle joints were affected.

There are many theories for the development of acute pseudogout following parathyroidectomy. The rapid drop in serum calcium decreases the solubility of CPPD, which can precipitate the shedding of crystals out of the cartilage and into the synovial fluid, inciting an inflammatory response. The phagocytosis of these crystals is the probable cause of provoking the acute inflammatory reaction within the involved joints [8]. Another proposed theory is that the sudden decrease in PTH affects phosphate metabolism by altering the equilibrium between the production of inorganic pyrophosphate (iPP) and its hydrolysis to orthophosphate. iPP, an integral part of CPPD crystal formation, increases the deposition of CPPD crystals. Phagocytosis of these crystals incites an acute inflammatory reaction within the joint [8].

The closest differential for calcium pyrophosphate disease is gout due to monosodium urate crystal deposition. Gout has an increasing prevalence with the rising obesity and metabolic syndrome pandemic [9]. Polarised microscopic examination of joint fluid enables a confirmatory diagnosis. The presence of positively birefringent rhomboid crystals suggests a diagnosis of CPPD, while negatively birefringent needle-shaped crystals are diagnostic of gout due to monosodium urate deposition [7]. Our patient had an elevated preoperative uric acid level, for which he was initiated on a xanthine oxidase inhibitor, febuxostat and had an improvement in serum uric acid levels on treatment. Any condition that alters extracellular urate levels can precipitate a gout flare. This includes surgical procedures, which could have been the inciting event in our patient. 

Few case reports exist reporting the simultaneous attack of gout and pseudogout in the same joint [10-12]. The presence of hyperuricemia followed by a surgical procedure, as well as severe PHPT-related bone disease and postoperative drop in calcium, could have been the trigger for the simultaneous gout and pseudogout in our patient. This has been proven by the presence of both these crystals in synovial fluid analysis. Kellet et al. [13] reported a 62-year-old female who presented with a history of podagra and hypercalcaemic crisis. Post-parathyroidectomy, she developed inflammation of the bilateral first metatarsophalangeal joints. Synovial fluid analysis confirmed deposits of calcium pyrophosphate as well as monosodium urate [13]. Hui et al. studied the association of serum uric acid with PTH levels among 8,316 participants of the National Health and Nutrition Examination Survey from 2003 to 2006 and noted that serum PTH levels are independently associated with uric acid levels [14]. In patients with PHPT, increased PTH levels are postulated to impair the transport and excretion of UA in the proximal renal tubules, resulting in hyperuricemia, although the precise mechanism is not yet fully understood [5].

The typical symptoms of pain and swelling of a joint in a patient operated for hyperparathyroidism should alert the physician to the possibility of acute crystal arthropathy. In view of the suggested associations of elevated uric acid with PHPT, it may be beneficial to monitor serum calcium, serum magnesium and serum uric acid in patients with acute inflammatory arthritis post-parathyroidectomy, as well as synovial fluid analysis to confirm the diagnosis.

## Conclusions

Although literature on the incidence of acute crystal arthropathy following parathyroidectomy is scarce, the presentation of acute joint pain and swelling should alert the clinician to this possibility. The presence of severe preoperative bone disease with elevated bone turnover markers should be an indicator of the possibility of such postoperative complications due to sudden shifts in serum calcium and phosphate levels. Mixed crystal arthropathy of monosodium urate and calcium pyrophosphate is rare. The concurrent presence of both crystal types in a single patient, though previously described in isolated case reports, seems to be linked to the combination of pre-existing hyperuricemia and postoperative calcium shifts, and this dual presentation necessitates heightened vigilance. Assessment using serum biochemical parameters as well as synovial fluid analysis plays a key role in reaching a diagnosis and initiating appropriate treatment for each of the underlying conditions. Physician awareness of acute crystal arthropathy as a postoperative complication of parathyroid surgery would aid in timely diagnosis and appropriate management. Further studies are needed to understand the associations between hyperparathyroidism and serum uric acid levels, as well as the optimal monitoring frequency and prognosis in dual crystal arthropathy. 
